# A Follow-Up Study of a European IgG4-Related Disease Cohort Treated with Rituximab

**DOI:** 10.3390/jcm10061329

**Published:** 2021-03-23

**Authors:** Johanna Backhus, Christian Neumann, Lukas Perkhofer, Lucas A Schulte, Benjamin Mayer, Thomas Seufferlein, Martin Müller, Alexander Kleger

**Affiliations:** 1 Department of Internal Medicine 1, Ulm University Hospital, 89081 Ulm, Germany; johanna.backhus@uniklinik-ulm.de (J.B.); christian.neumann2@googlemail.com (C.N.); lukas.perkhofer@uniklinik-ulm.de (L.P.); luas-alexander.schulte@uniklinik-ulm.de (L.A.S.); thomas.seufferlein@uniklinik-ulm.de (T.S.); 2 Institute of Epidemiology and Medical Biometry, Ulm University, 89075 Ulm, Germany; benjamin.mayer@uni-ulm.de

**Keywords:** IgG4-related disease, autoimmune pancreatitis, rituximab, Responder Index

## Abstract

Objectives: IgG4-related disease (IgG4-RD) is a chronic fibro-inflammatory disorder affecting virtually any organ. Type 1 autoimmune (type 1 AIP) is its pancreatic manifestation. To date, steroids are considered the first-line pancreatitis treatment. The CD20-binding antibody rituximab (RTX) appears a promising steroid-sparing therapy, although long-term data are lacking. We aimed to bridge this gap with a cohort of IgG4-RD patients treated with RTX and to assess the potential value of the Responder Index (RI) as a discriminatory score for disease activity. Methods: We retrospectively evaluated 46 patients from a tertiary referral centre who were diagnosed with IgG4-RD and/or type 1 AIP according to the International Consensus Diagnostic Criteria or Unifying-AIP criteria between June 2006 and August 2019. Results: Patients resembled previous cohorts in terms of characteristics, diagnosis, and therapeutic response. Thirteen of the 46 patients with IgG4-RD/type 1 AIP were treated with RTX pulse therapy due to relapse, adverse reactions to steroids, or high-risk constellations predicting a severe course of disease with multi-organ involvement. Median follow-up after diagnosis was 52 months for all subjects, and 71 months in IgG4-RD patients treated with RTX. While patients in the RTX group showed no significant response to an initial steroid pulse, clinical activity as measured by the RI significantly decreased in the short-term after RTX induction. Within 16 months, 61% of patients relapsed in the RTX group but responded well to re-induction. Clinical and laboratory parameters improved equally in response to RTX. Conclusion: RTX therapy in patients with IgG4-RD is an effective and safe treatment to induce treatment response and possible long-term remission. Repeated RTX administration after 6–9 months may be of value in reducing the risk of relapse. The RI appears to be a reasonable index to assess disease activity and to identify patients with IgG4-related disease who may benefit from B-cell-depleting therapy.

## 1. Introduction

In 1961, autoimmune pancreatitis (AIP) was first described as an independent entity of chronic pancreatitis [1]. Two subtypes have been defined: (i) type 1 AIP, as part of IgG4-related disease and (ii) type 2 AIP. The International Consensus Diagnostic Criteria (ICDC) [2] and the Unifying-Autoimmune-Pancreatitis-Criteria (U-AIP) [3] serve as established scoring systems to evaluate both clinical probability and the specific subtype of AIP. Criteria include imaging, histology, serology, other organ involvement, and steroid response. The American College of Rheumatology (ACR)/European League Against Rheumatism (EULAR) recently presented new IgG4-RD classification criteria with an excellent test performance. They include 11 possibly affected organs and 32 clinical, serological, radiological and pathological items [4,5]. IgG4-RD is frequently associated with high levels of IgG4, multi-organ involvement including cholangitis or nephritis, and histopathological findings of lymphoplasmacytic sclerosing inflammation [6]. In the absence of contraindications, steroids are the recommended first-line treatment in all patients [7,8]. The initial response rate to steroids is usually more than 92% [9,10], but relapse rates may be as high as 50%. Moreover, the adverse effects of repeated steroid pulses can become clinically relevant [11] Meanwhile, rituximab (RTX)—as a B-cell-depleting antibody targeting CD20 surface antigen—represents an established step-up therapy with favourable safety and efficacy profiles. RTX is, therefore, already included in the new European Guideline on IgG4-related digestive disease [8,12] However, long-term data on RTX in IgG4-RD are still limited. Specific clinical indicators to decide which patients require RTX, its dosing intervals, and, especially, long-term efficacy data remain elusive [13]. The Responder Index (RI) is a modification of the Birmingham Vasculitis Activity Score for Wegener’s granulomatosis, which was initially developed and validated to assess clinical activity and organ damage and has subsequently been used for rating treatment efficacy [14,15]. Current knowledge indicates that serum IgG4 levels prior to steroid therapy are frequently not available and have several shortcomings as a disease marker [16,17,18]. Therefore, serum IgG4 levels per se are a poor predictor of relapse after steroid therapy [10], although high levels indicate an aggressive course of disease with multi-organ involvement [16]. A recent post-hoc analysis suggests that physical global assessment correlated better with the RI when serum IgG4 levels were omitted, leading to revision of the RI [15,19]. In turn, we also employed the modified RI without serum IgG4 levels in order to identify specific disease subgroups in the current study. Plasmablasts found in the peripheral blood, which are precursors of tissue-resident, antibody-producing plasma cells, play a key role in the pathophysiology of IgG4-associated diseases and correlate with disease activity better than serum IgG4 levels. The effects of RTX are also linked to the depletion of cell descendants of B lymphocyte-like plasmablasts [20].

The aims of this analysis were to examine the outcome in patients with IgG4-RD and type 1 AIP, focusing on the long-term follow-up of a subgroup of IgG4-RD subjects on RTX therapy in a real-life setting from a pancreatic outpatient unit at a German tertiary referral centre, and to test the RI for its ability to discriminate disease activity after distinct treatments.

## 2. Patients and Methods

### 2.1. Inclusion/Exclusion Criteria

All patients (*n* = 46) diagnosed with IgG4-RD/type 1 AIP treated in a dedicated pancreatic outpatient unit at Ulm University Hospital between June 2006 and August 2019 were included in this retrospective analysis. Follow-up continued until November 2020. Diagnostic criteria for inclusion were either probable or definite positivity according to ICDC [2] and/or positivity according to U-AIP [3]. We excluded patients who did not meet the ICDC or U-AIP criteria. ACR/EULAR classification criteria were also used [4,5], but were not decisive for inclusion or exclusion. Data were obtained retrospectively and pseudonymization was performed upon initial data collection in accordance with the Helsinki Declaration. The analysis was reviewed and approved by the local ethics committee (No. 364/17, 16.10.2017, Ulm University).

### 2.2. Clinical and Morphologic Data

Demographic, clinical, laboratory, histological and radiological characteristics were collected retrospectively for all patients (Table 1 and Table 2). For further details, please refer to the Supplementary Results and Methods and Supplementary Figure S1.

### 2.3. Responder Index (RI)

The modified RI was calculated to evaluate the activity of the disease at three timepoints, as described previously in [15]: before steroid therapy, before RTX therapy, and at the last visit to our clinic after any kind of treatment. The score includes the absolute number and severity of organs affected, clinical symptoms, urgency of treatment, and presence of organ dysfunction. The score distinguishes between “symptoms” and “permanent organ damage” to differentiate between acute inflammatory activity and irreversible dysfunction. Each affected organ generates points for disease activity: zero points for “unaffected” or “resolved”, one point for “improved but persistent”, two points for “new or recurrence (while off treatment) or unchanged”, and three points for “worse or new despite treatment”. If any organ requires treatment immediately to prevent serious organ dysfunction, the points scored by this organ are doubled. In addition, we assessed organ damage, stable remission, and relapse-free time. “Damage” means organ dysfunction that has occurred as a result of IgG4-RD and is considered permanent. “Stable remission” means a decrease of the RI to a level with zero activity after treatment, with or without steroid maintenance therapy. Please refer to the Supplemantary Table S1 for more details on the score.

### 2.4. Treatment Protocols

**Figure 1:**
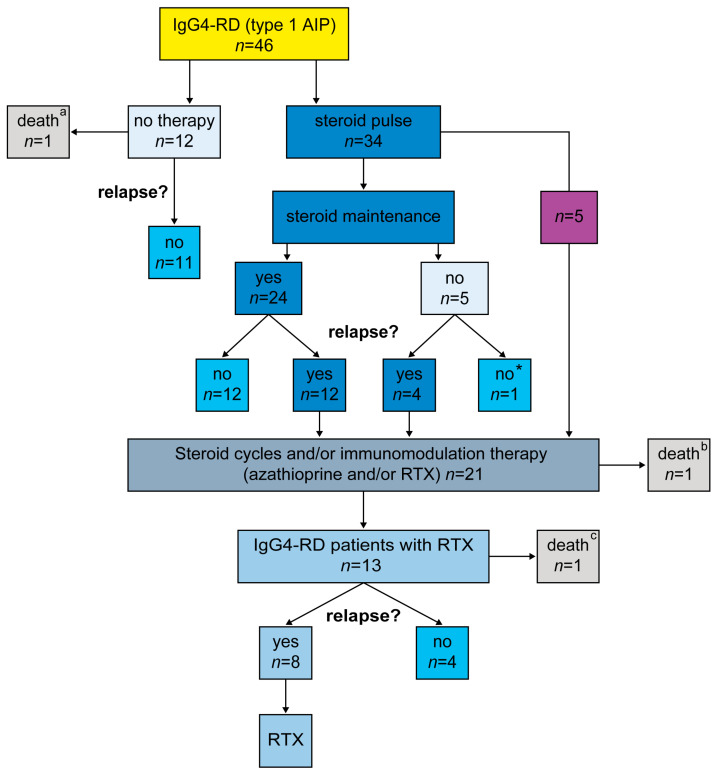
Diagram showing different treatment regimens in patients with IgG4-RD. IgG4-RD: IgG4-related disease; RTX: rituximab; * a patient lost to follow-up after steroid pulse therapy; ^a^ a patient with histologically confirmed IgG4-RD not on medication, who died from complications after surgery for suspected cancer; ^b^ a patient treated after relapse with steroids, who died from intraductal papillary mucinous neoplasm that progressed to pancreatic cancer; ^c^ a patient who had been treated with steroid pulse therapy and RTX (as a last resort) but died from sepsis.

Detailed treatment regimens are presented in Figure 1 and Table 2.

Steroids: Steroid therapy was initiated in patients with symptomatic acute or chronic IgG4-RD/type 1 AIP, including jaundice and/or confirmed multi-organ involvement. Treatment was usually initiated with 40 mg for 4 weeks, followed by tapering the dose and maintenance therapy of 2.5–7.5 mg/day. As their treatment had been initiated in non-specialised centres before attending our outpatient unit, some patients received higher initial steroid dosages [8,21].

Immunosuppression: Azathioprine maintenance therapy was administered at dosages of 2–2.5 mg/kg/day.

*Rituximab:* RTX maintenance therapy was administered after steroid induction for (i) steroid-dependent IgG4-RD, (ii) relapse, or (iii) severe multi-organ involvement. RTX was originally administered at a dosage of 375 mg/m^2^ four times at weekly intervals [22] but then routine practice changed to an absolute dose of 1000 mg on two occasions 14 days apart [19]. All patients received premedication with (iv) methylprednisolone (100 mg), dimetindene (4 mg), and ranitidine (50 mg) before RTX therapy. In the case of persistent minimal disease activity, some patients were prescribed steroids of 2.5–5 mg/day in addition to the RTX cycles. Other immunosuppressive therapies were stopped before RTX treatment. Patients were screened for hepatitis B and C, HIV, and tuberculosis before initiating treatment with RTX.

## 3. Results

### 3.1. Baseline Characteristics

Forty-six patients, consisting of 31 men (67%) and 15 women (33%), were included in the analysis. The mean age was 54 years (SD ± 16, range 21–87) (Table 1). For more information regarding the characteristics of the study population, diagnostic criteria, serology, and complications, see Table 1 and the Supplementary Results.

### 3.2. Treatment Response and Relapse Rates

In accordance with the current guidelines, 74% (34/46) of IgG4-RD patients were initially treated with a steroid pulse. The response rate after the initial steroid pulse was 97% (33/34). Twelve patients (26%) were not treated, due to their being in remission at the time they presented to our centre (Table 2).

Three patients (6%) died during follow-up: one from pancreatic cancer, one from sepsis, and one after pancreatic surgery. One patient was lost to follow-up.

Based on disease progression and initial treatment regimens (Table 2) we identified three groups: group 1 received a steroid pulse followed by low-dose steroid maintenance therapy and included 24 (71%) of the 34 IgG4-RD patients. The five patients (15%) forming group 2 received no maintenance therapy. The five (15%) forming group 3 received immunomodulatory maintenance therapy, with the initial steroid pulse directly followed by either azathioprine (*n* = 3) or rituximab (*n* = 2), mostly due to steroid dependence or severe multi-organ involvement (see next section on RTX patients, Figure 1).

We observed clinical relapse in 20 (43%) of the entire 46 patients. The first relapse occurred after a median of 12 months, irrespective of therapy. In group 1, 50% (12/24) patients suffered a relapse after a median of nine months. In contrast, 80% (4/5) patients in group 2 had a relapse after a median of ten months (*p* = 0.3432). Finally, in group 3, three patients on azathioprine (100%) and one on rituximab (50%, see next section on RTX) relapsed. The median time to relapse was 36 months in the azathioprine subgroup.

Relapse treatment options included additional steroid pulses, azathioprine, azathioprine followed by RTX, or RTX (see also next section, Table 2).

### 3.3. Treating IgG4-RD Patients with Rituximab

**Figure 2:**
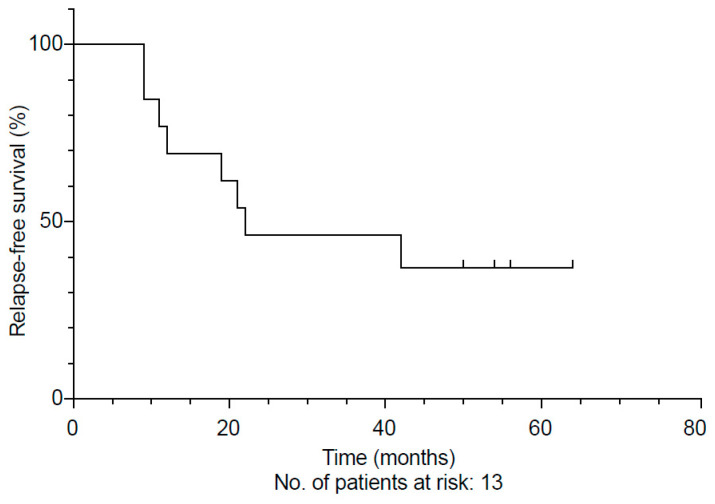
Onset of clinical relapse in 13 patients treated with rituximab (RTX), displayed as a Kaplan–Meier curve.

Intravenous RTX regimens consisted of either four doses of 375 mg/m^2^/week in 92% or two doses of 1000 mg at an interval of 14 days in 54%, following regimens reported previously [19,22]. In total, 13 patients with IgG4-RD were treated with RTX at our centre. The median follow-up was 71 months (range 2–173 months) from initial diagnosis and 51 months (range 1–71 months) from the start of RTX therapy. Overall, the relapse rate in this subgroup was 61% (8/13). The median relapse-free survival after RTX was 16 months (Supplementary Table S2). Half of these patients had a clinical relapse within 21 months (Figure 2). Possible adverse events during treatment with RTX included a varicella-zoster infection (*n* = 1), atypical pneumonia (*n* = 1), and a post-operative pulmonary embolism. In addition, one patient with highly aggressive IgG4-RD died from acute cholangiosepsis and pneumonia followed by multi-organ-failure after a high-dose steroid pulse and RTX had been given as a last resort.

**Figure 3:**
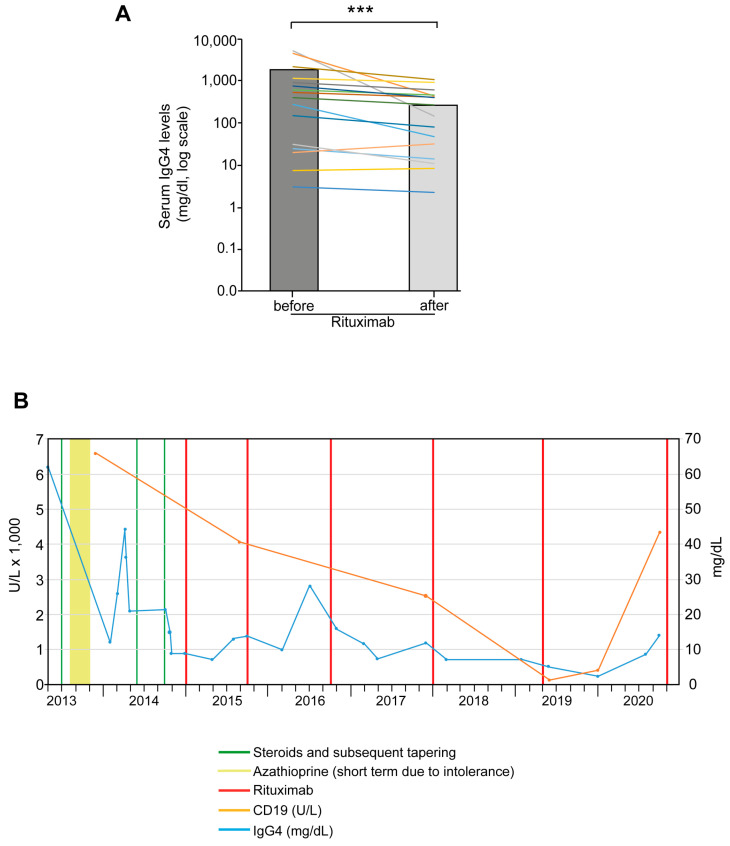
Course of serological markers after RTX therapy. (**A**) Significant decrease in serum IgG4 levels in patients treated with RTX. Bars indicate mean IgG4 levels before and after treatment. Coloured lines show individual development of serum IgG4; *** *p* < 0.001. (**B**) Representative Illustration of changes in CD19 cell count (orange line) and IgG4 levels (blue line) in an individual patient after steroid pulses (green vertical lines) and several administrations of RTX (red vertical lines). Peak doses of the three steroid pulses were 60, 25, and 30 mg, respectively. The light green bar represents treatment with azathioprine for 4 months.

Serum IgG4 levels were monitored throughout treatment and showed a significant decrease after RTX therapy (*p* < 0.001). Figure 3A shows the serum IgG4 levels of each patient before and after treatment with RTX. To illustrate the therapeutic response based on CD19-positive B lymphocytes before and after RTX, we present one patient as an example: Figure 3B shows the clinical course of this patient with IgG4-RD affecting multiple organs. At first contact, he had pancreaticobiliary, liver, renal and lymph node involvement, with a serum IgG4 level of 6200 mg/dL and an increased number of CD19^+^ cells (47/µL). After the initial steroid pulse therapy had been tapered off, azathioprine was used as maintenance therapy but stopped early due to intolerance. Because of persistent steroid-dependence and frequent steroid pulses, RTX was initiated and administered repeatedly, finally leading to sustained remission with consistently lowered IgG4 and CD19^+^ levels. Overall, univariable analysis did not reveal any correlation of clinical or serological parameters with relapse-free survival after maintenance therapy with RTX (Supplementary Table S3).

### 3.4. Assessing Treatment Response According to the Responder Index

**Figure 4:**
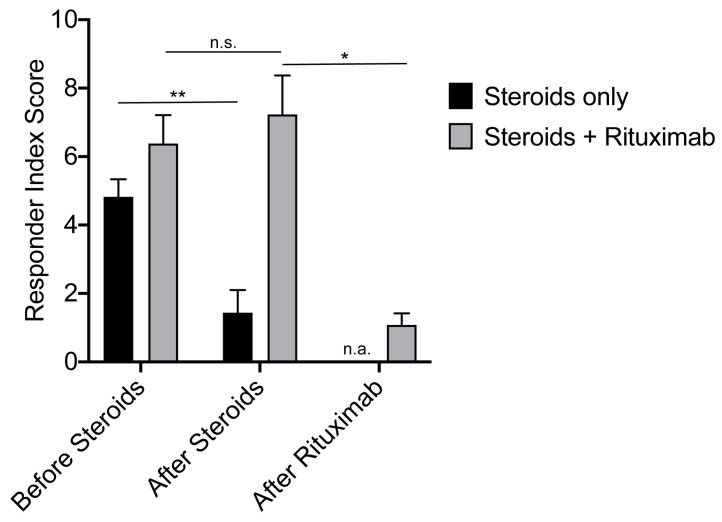
Treatment response, measured by the RI in patients treated either with steroids or with steroids and subsequent RTX. RI scores were evaluated (i) before the initiation of steroid therapy, (ii) after the termination of steroids and (iii) after RTX therapy; * *p* < 0.05, ** *p* < 0.01, n.s. not significant.

To employ the clinical value of the RI in our study cohort, we looked at all IgG4-RD patients whose disease was treated with steroids alone, even during relapse (“Steroids” subgroup; *n* = 17, one patient died). Interestingly, the mean RI for the “Steroids” subgroup was 4.8 before treatment and fell to 1.4 during follow-up. Stable remission was achieved in 11 patients (65%). The second subgroup comprised all patients who were initially treated with steroids followed by RTX for disease control, either initially or during relapse (“RTX” subgroup; *n* = 13). In the latter, the RI of 6.4 before steroid therapy was in a similar range to the “Steroids” subgroup, indicating overall homogeneous patient demographics across the two groups. After the steroid pulse, the RI in the “RTX” subgroup remained high at 7.2. However, with RTX treatment the RI dropped significantly to 1.1 at the last visit (Figure 4). Stable remission during the entire follow-up period was achieved in four patients (31%) on RTX. Even though the RI could discriminate patients with a steroid response from a refractory subgroup, it showed no correlation according to clinical or serologic parameters with the last visit to our clinic (Supplementary Table S3).

## 4. Discussion

IgG4-RD is a chronic fibro-inflammatory disorder affecting virtually any organ. It is characterised by exceptionally high response rates to a steroid pulse. However, a relevant number of patients, usually characterised by high serum IgG4 levels and/or multi-organ involvement, present with either frequent relapses or steroid dependence before reaching remission. Thus, steroid-sparing immunomodulatory therapy is becoming a cornerstone of treatment in these patients. While previous studies have proposed established disease-modifying antirheumatic drugs (DMARDs) such as azathioprine, recent data suggest more precise interventions, such as the B-cell-depleting agent RTX, to interrupt the pathophysiological cascade of events in IgG4-RD [17,23]. While data on RTX treatment response are becoming more frequent, follow-up data on remission and relapse rates on RTX therapy remain sparse [22,24,25]. With our study, we bridge this gap in the knowledge and present a European IgG4-RD cohort of 46 patients initially treated with steroids who received treatment with RTX when meeting the criteria of (i) steroid-dependent IgG4-RD, (ii) relapse, or (iii) severe multi-organ involvement. The median follow-up time was 52 months but ranged up to 71 months. In addition, we employed the RI as a recently developed clinical, serological, and radiological composite disease-monitoring score to separate patients requiring RTX from those who could easily be controlled by steroids alone.

Plasmablasts express CD19^low^CD20^−^CD27^+^CD38^+^ and are believed to be the precursors of tissue-resident, antibody-producing plasma cells. In this context, the effects of RTX are worthy of note, since the depletion of B cells results in the successful elimination of plasmablasts [20,24,26]. However, B cells are not the only driving force in IgG4 pathophysiology: the detection of oligoclonal cytotoxic T cell (CTL) populations such as CD4-positive CTLs correlates well with disease activity, but again, there is relevant cytokine crosstalk to the B cell compartment [24,27]. Several studies, including an open label trial, have suggested that B cell depletion with RTX is an effective treatment for IgG4-RD [12,19,22]. Accordingly, RTX is now part of the new guidelines for the second-line treatment of IgG4-RD. Specifically, it is recommended after insufficient steroid therapy or as third-line treatment after the failure of other immunomodulators such as azathioprine [2,8,12,25]. However, there are scant follow-up data on RTX-treated IgG4-RD real-life cohorts. Campochiaro et al. treated 14 patients with RTX and followed them up for 18 months [13], while another study followed 33 patients for 25 months [25]. Even so, the question of the optimal administration regimen and clear recommendations for treating relapse with RTX have not yet been fully addressed [8]. It appears that relapse-free survival time depends on the frequency of RTX treatment, as a fixed dosing interval of every 6 months can eventually prevent relapse [13]. Similarly, repeated RTX doses at varying intervals prolonged the relapse-free survival probability. In our study, 8 of 13 patients (61%) treated with RTX relapsed. The overall relapse rate remained superior to that of azathioprine-treated individuals, although the case load with the latter treatment remained low (three out of three patients). In our cohort, relapse occurred within 16 months of the first dose of RTX; repeat treatment was given on signs of relapse and not pre-emptively, in order to avoid overtreatment. Most of our patients received an induction regimen of 375 mg/m^2^ weekly four times according to initial reports [22] but our routine practice switched to 1000 mg two times at an interval of 14 days when the results from the first open-label trial were reported [19]. Several studies reported other organ, proximal bile duct involvement or steroid treatment as predictive for relapse per se, independent of the treatment [8,12,28]. In contrast, in our study a univariable analysis focusing only on relapsing vs. non-relapsing patients receiving rituximab treatment, such factors did not have predictive power, as reported before [12,28]. However, a higher sample size of the rituximab-treated population and measurement of additional biomarkers including e.g., plasmablasts might help to identify such predictive factors for RTX-treated patients as well. Some patients with IgG4-RD in our cohort underwent surgery for suspected cancer but, as expected, surgery did not prevent relapse (82%, Table 2). However, other authors have reported a lower relapse rates after surgery [29]. Surgery in our cohort was mostly performed in other facilities without administration of the currently recommended two-week steroid therapy [8,30], a fact potentially promoting higher relapse rates.

Clinical response in the 13 IgG4-RD patients who received RTX for maintenance therapy after a steroid pulse was evaluated by the Responder Index and clinical assessment including imaging. The RI is currently the only validated score for assessing IgG4-RD activity to standardise clinical outcome measures. It shows a high correlation with the response to RTX therapy [15,25,31]. Both real-life experience and the current literature indicate that serum IgG4 levels prior to steroid therapy are frequently lacking and that serum IgG4 levels have shortcomings as a disease marker [16,17,18]. The modified RI [15] showed a significant reduction in the steroid-only subgroup, but there was no such decrease after steroids in the subgroup later treated with RTX, indicating the insufficient steroid response. In contrast, RTX treatment significantly reduced the RI to within the range of patients adequately treated with steroids alone. Recently, a study applied the first-generation Responder Index to their study cohort, treated with rituximab two and four months after induction [14,32]. Although this version of the Responder Index is slightly different to the revised RI [15], due to the higher priority given to “active disease”, the results were similar to ours. Specifically, relapse rate correlated with higher scores after induction, indicating that insufficient RI decline could help guide relapse therapy even after RTX treatment. Similar data were reported by others licensing the RI as a putative treatment guidance instrument of IgG4-related disease [25,31]. In our study, we did not see such a correlation, but the RI assessment before and after steroid pulse therapy allows us to identify a subgroup potentially benefitting from RTX.

Steroids remain the cornerstone of first-line treatment for IgG4-RD [8,21] with a primary response of up to 99%; the response to steroids is even used as a diagnostic criterion [3,8]. However, relapse rates are also high, particularly in the first three years and while tapering the steroid dose [10,22,23,24,33]. Therefore, in our study, most patients received a steroid pulse followed by tapering and maintenance therapy, since a randomised controlled trial had reported fewer relapses with this regimen [9]. However, some patients either refused the maintenance treatment due to fear of either steroids or the exacerbation of pre-existing diabetes. These patients and those requiring steroid dosages above the Cushing threshold, therefore, received either azathioprine or RTX. In line with previous studies, all patients treated with azathioprine relapsed [34,35]. Thus, alternative steroid-sparing agents beyond classical DMARDs are warranted. In the future, novel concepts may be even more personalised. For example, XmAb5871, an antibody targeting the plasmablast surface marker CD19, might add to the current B-cell-depleting agent repertoire [36]. Serum IgG4 levels remain a diagnostic hallmark for IgG4-RD [2], although a poor diagnostic performance has been reported, particularly in non-Asian populations. In our study, 54% of the entire cohort presented with elevated serum IgG4 levels, underlining this diagnostic weakness also in the European population [16,17,18,23]. Even so, serum IgG4 levels in our patients declined during clinical response, particularly with RTX treatment, qualifying serum IgG4 to monitor RTX treatment in the cases where levels are elevated beforehand. In line with plasmablasts being CD19^low^-positive and a valid biomarker for IgG4-RD [17], we measured the CD19-positive cell population in some patients before and after RTX therapy and observed a downregulation (data not shown). However, more systematic examination would have been necessary to draw any definite conclusions.

Besides serological biomarkers, bioactive imaging such as PET-CT has been suggested for assessing disease activity properly and distinguishing it from fibrotic damage in IgG4-RD. Although large cohorts have demonstrated the efficacy of PET-CT [37,38] and the new guidelines underline its value [8], we did not systematically evaluate this technique in our cohort. However, PET scans provided significantly more insight into activity patterns than conventional imaging techniques, particularly in the case of large vessel involvement, but the low case numbers in our study prevented any firm conclusions. Future studies are needed to assess its value in light of the organs involved.

Our study inclusion criteria were based on either the ICDC or U-AIP classification. Interestingly, by using the new ACR/EULAR classification, we would have lost many patients, as it identified only 37%. The large number of patients with type 1 AIP, in whom obtaining a tru-cut needle biopsy specimen remains challenging, is most likely responsible for this diagnostic gap, as this classification strongly relies on histological criteria [4,5]. However, more studies are warranted to examine this explanation in other European cohorts. The mean age of our patients was slightly lower than in previous reports [39]. Exocrine pancreatic insufficiency, present in 24% of our IgG4 cohort, is also in agreement with the large number of type 1 AIPs, as one-third of these patients develop pancreatic atrophy and subsequent insufficiency within three years of diagnosis [40]. Endocrine insufficiency rates, gender distribution, and cross-organ manifestations also reflect previous reports. Unfortunately, the limited measurements of pancreatic function during the course of disease prevented us from putting treatment response into direct correlation. A high proportion of our patients (65%) had multi-organ involvement (median two organs affected; range 1–5), again a feature validating the representativeness of our cohort [41]. However, lymphadenopathy was frequently lower in our cohort than in others, although most patients in our study underwent complementary imaging techniques [25]. Overall, our cohort appears to be representative of the European IgG4-RD population, hence allowing our conclusions.

### Strengths and Limitations

The main weakness of our study lies its retrospective design, which limits firm conclusions on therapeutic recommendations. However, our study is one of only a few reporting true long-term, follow-up data in a representative European IgG4-RD cohort treated with RTX [13,25]. As in other reports [13,25], our study cannot address the extent to which RTX therapy prevents organ damage, which is mostly driven by the fibrotic reaction present in IgG4-RD. Future studies are warranted to address this critical concern. Another problem is the lack of a standardised RTX treatment protocol, although we switched to an absolute dosage 1 g at our centre following the work of Carruthers et al. [19]. Similarly, steroid maintenance therapy was not homogeneously administered to all patients. Finally, the case numbers in our study remain low, despite our cohort being one of the largest in Europe connected to a single centre [42]. Multicentre studies, such as the planned PrescrAIP initiative, which will present the pan-Europe experience on current AIP treatment regimens [42], are urgently needed. A randomised controlled setting focused on RTX, like that achieved by the trial on long-term maintenance corticosteroid therapy [9], should be a future goal to harmonise the treatment of IgG4-RD.

## 5. Conclusions

RTX maintenance therapy should be considered only for difficult-to-treat IgG4-RD patients, but seems to be safe and effective. Relapse remains common but repeated RTX administration is reasonable and feasible to control disease. The RI is a valid index to assess disease activity and identify patients who would potentially benefit from a B-cell-depletion strategy.

## Figures and Tables

**Table 1:** Characteristics of patients with IgG4-related disease at baseline. Exocrine and endocrine insufficiency during follow-up.

Characteristic	IgG4-RD (*n* = 46)
**Mean age—years**	54.2
**Sex—number (%)**	
male	31 (67.4)
female	15 (32.6)
**Smoking status—number (%)**	
active	13 (28.3)
never	20 (43.5)
former	13 (28.3)
Exocrine insufficiency—number (%) *	
yes	11 (26.8)
no	27 (65.8)
unclear	3 (7.3)
Endocrine insufficiency—number (%) *	
yes	12 (29.3)
no	28 (68.3)
unclear	1 (2.4)

* only patients with pancreatic involvement (*n* = 41).

**Table 2:** Clinical and therapeutic aspects of patients with IgG4-related disease.

Characteristic	IgG4-RD (*n* = 46)	
**Extrapancreatic manifestation—number (%)**		
yes	30 (65.2)	
no	16 (34.8)	
**Number of affected organs—mean (min; max)**	2 (1; 5)	
		
**Steroid pulse—number (%)**		
yes	34 (73.9)	
no	12 (26.1)	
		
**Steroid maintenance therapy—number (%)**		
yes	24 (82.8)	
no	5 (17.2)	
		
**Relapse therapy**	IgG4-RD (*n* = 20)	
**Steroids + immunomodulatory therapy—number (%)**		
azathioprine mono	3 (15)	
azathioprine followed by rituximab	5 (25)	
rituximab mono	7 (35)	
**Steroid cycle therapy—number (%)**	5 (25)	
		
**Surgery—number (%)**	**IgG4-RD (*n* = 11)**	**Relapse (*n* = 9)**
pancreatectomy	1 (9.1)	0
partial pancreatic resection	6 (54.5) *	5 (55.6)
partial nephrectomy	1 (9.1)	1 (11.1)
submandibulectomy	2 (18.2)	2 (22.2)
tumor resection at the pulmonary artery	1 (9.1)	1 (11.1)

* one patient died after surgery.

## Data Availability

All data relevant to the study are included in the article or uploaded as supplementary information.

## Supplementary Materials

The following are available online at https://www.mdpi.com/2077-0383/10/6/1329/s1, Figure S1: Frequency of distinct organ manifestations in male and female patients with IgG4-RD (possible mention of several organs per patient). Table S1: Scoring parameters and clinical definitions of the Responder Index (RI). Table S2: Responder Index—clinical and therapeutic aspects of the “Steroids” and “RTX” subgroups. Table S3: Univariable analyses of modifier and time to relapse or Responder Index at the latest point in time in IgG4-RD patients with RTX treatment. Estimate: 95%CI.
