# Supplementary material for: A Follow-Up Study of a European IgG4-Related Disease Cohort Treated with Rituximab
Source: J Clin Med. 2021 Mar 23;10(6):1329. doi: 10.3390/jcm10061329 (PMC8004657; doi:10.3390/jcm10061329)
Supplement: Supplementary file 1 [file jcm-10-01329-s001.zip › Backhus et al._IgG4-RD_06.03.2021_Supplementary results, methods and figure.docx]

**Supplementary data set:**

**Supplementary Results**

**Baseline characteristics**

IgG4-RD patients (n=46) presented with distinct organ manifestation patterns, dominated by type 1 AIP (n=41; 89%). Type 1 AIP was frequently associated with immune-associated cholangitis (n=11; 24%). Only 16 (35%) patients with IgG4-RD showed disease activity exclusively in the pancreas, while 30 (65%) patients presented with multi-organ involvement. On average, two organs were affected (maximum of five) (**Table 2**). In descending order, the organs most affected were the pancreas, bile duct, lymph nodes, kidney, and liver **(Supplementary Figure 1)**. Smoking status showed similar numbers of active and former smokers. There were twice as many who had never smoked (**Table 1**). Median follow-up after diagnosis was 52 months for all subjects.

**Diagnostic criteria and serology**

Seventy-six per cent of all patients (n=35/46) fulfilled ICDC criteria and 87% (n=40/46) met U-AIP criteria. ACR/EULAR classification criteria identified 37% (n=17/46). Twenty-eight of the 46 IgG4-RD patients (61%) had either a biopsy (n=15; 33%), underwent surgery to retrieve tissue specimens (n=2; 4%) or histology was performed because of surgery for suspected cancer (n=11; 24%). Histology was obtained in 28 patients, but only 16 patients fulfilled histological criteria with IgG4 expression above the cut-off.

Nine out of 11 patients (82%) who underwent surgery for suspected cancer experienced a relapse during follow-up.

Serum IgG4 levels were measured in 41 out of 46 treatment-naïve IgG4-RD patients: they were elevated in 54% (22/41) and twice the upper-limit of normal in 37% (15/41). Serum IgE levels were available in 22 patients: they were elevated in 73% (n=16). ANA titres were above normal limits in 20 out of 34 patients (59%).

**Complications**

We evaluated exocrine and endocrine insufficiency as potential complications of AIP: ten (24%) of 41 patients with type 1 AIP were either initially diagnosed with or later developed exocrine insufficiency (mean follow-up 52 months). More than one in four (12/41; 29%) had an endocrine insufficiency due to complications of the disease or while on treatment **(Table 1)**.

**Supplementary Material and Methods**

**Diagnostic definitions**

Exocrine insufficiency was defined as low faecal elastase <200 µg/g, endocrine insufficiency by HbA1c >6.5 g/dL. Smoking status was assessed at different points in time. Detailed data was obtained for all patients with type 1 AIP with or without other organ involvement. Other organ involvement, defined as extrapancreatic manifestation, was confirmed by radiological criteria (MRI, endosonography, CT), histological findings, or past medical history with a pathognomonic presentation. The radiological diagnosis of AIP was confirmed by typical findings on imaging such as diffuse (“sausage-like”) or focal swelling of the pancreas or signs of chronic pancreatitis. Start and stop dates were recorded for steroids and other immunosuppressive treatment such as azathioprine. Whenever possible we determined established serum markers. Serum IgG4 levels were measured in patients on RTX therapy before and after treatment. B lymphocytes with CD19 expression were determined as a parameter of B cell depletion in a few patients with IgG4-RD treated by RTX.

**Assessment of treatment response and disease relapse**

Response was defined by a decrease of clinical symptoms combined with radiological signs. Relapse was defined as the reappearance of clinical symptoms and signs (such as pancreatic pain, cholestasis, and swollen lymph nodes) or findings on imaging (inflammation of the vessels) or abnormal laboratory test results (elevated levels of IgG4 or liver enzymes).

**Statistical analysis**

Study data was analysed descriptively using the mean, standard deviation (SD), and range in the case of quantitative parameters, and using frequencies and percentages for categorical variables. The Fisher exact test was used to compare frequencies. A possible correlation of several clinical predictors with both RI and relapse-free survival after steroid or RTX treatment was determined by means of linear regression and Cox proportional hazard regression models, respectively. An explorative two-sided p‑value ≤ 5% was considered statistically significant. We used the Wilcoxon matched-pairs signed rank test and the R software for statistical computing (version 3.5.1, [www.r-project.org](http://www.r-project.org)) for the paired comparisons (*p<0.05, **p<0.01, ***p=0.001, n.s. not significant). Follow-up times were always reported as the median.

**Supplementary Figure**

**Suppl. Figure 1:** Frequency of distinct organ manifestations in male and female patients with IgG4-RD (possible mention of several organs per patient).
