# Supplementary figures and images for: A Follow-Up Study of a European IgG4-Related Disease Cohort Treated with Rituximab
Source: J Clin Med. 2021 Mar 23;10(6):1329. doi: 10.3390/jcm10061329 (PMC8004657; doi:10.3390/jcm10061329)

Supplemental Figure 1

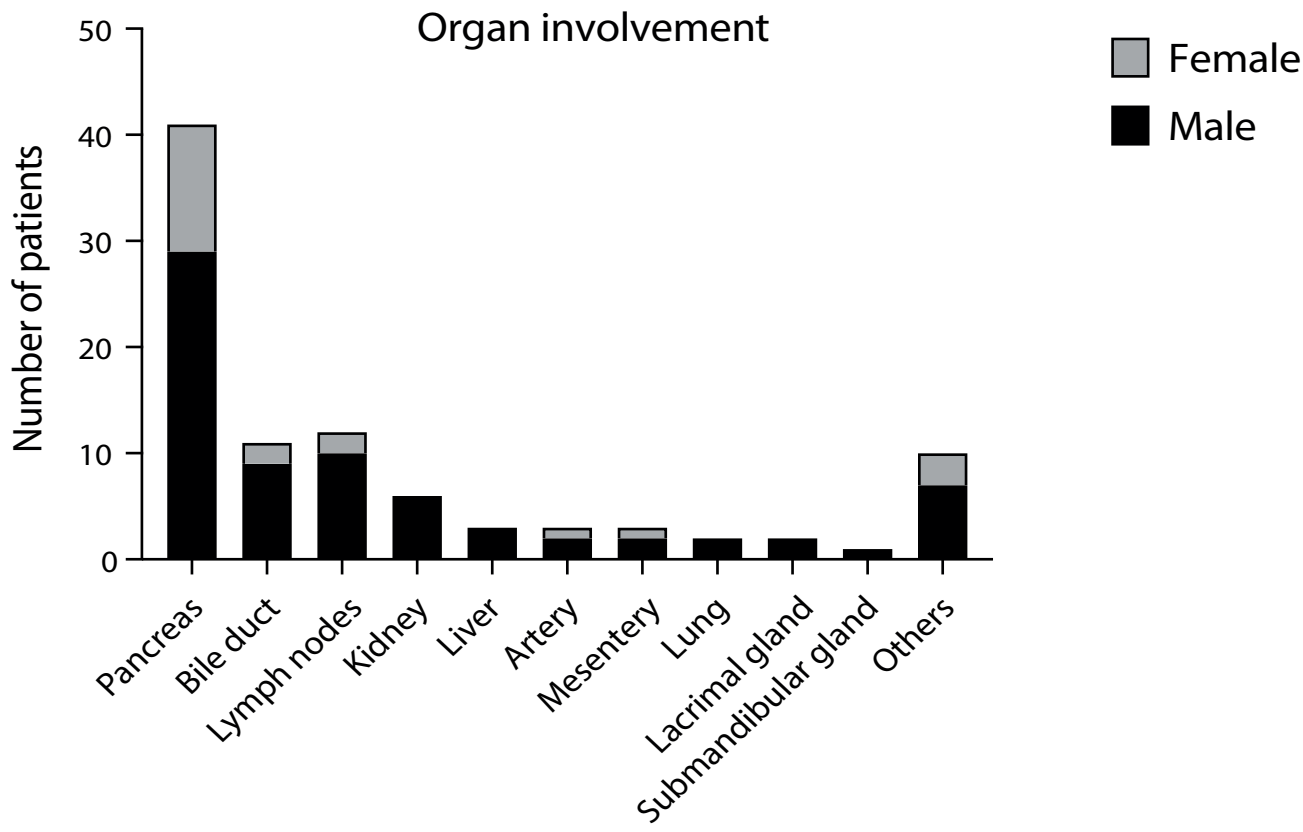

Supplement: Supplementary file 1 [file jcm-10-01329-s001.zip › Suppl Figure 1_10.02.2021.pdf]
